# First‐Principle‐Based Phonon Transport Properties of Nanoscale Graphene Grain Boundaries

**DOI:** 10.1002/advs.201700365

**Published:** 2018-01-11

**Authors:** Leonardo Medrano Sandonas, Hâldun Sevinçli, Rafael Gutierrez, Gianaurelio Cuniberti

**Affiliations:** ^1^ Institute for Materials Science and Max Bergmann Center of Biomaterials TU Dresden 01062 Dresden Germany; ^2^ Max Planck Institute for the Physics of Complex Systems 01187 Dresden Germany; ^3^ Department of Materials Science and Engineering Izmir Institute of Technology 35430 Urla Izmir Turkey; ^4^ ICTP‐ECAR Eurasian Centre for Advanced Research Izmir Institute of Technology 35430 Urla Izmir Turkey; ^5^ Dresden Center for Computational Materials Science (DCMS) TU Dresden 01062 Dresden Germany; ^6^ Center for Advancing Electronics Dresden TU Dresden 01062 Dresden Germany

**Keywords:** DFTB calculations, grain boundaries, graphene, Landauer theory, phonon transport

## Abstract

The integrity of phonon transport properties of large graphene (linear and curved) grain boundaries (GBs) is investigated under the influence of structural and dynamical disorder. To do this, density functional tight‐binding (DFTB) method is combined with atomistic Green's function technique. The results show that curved GBs have lower thermal conductance than linear GBs. Its magnitude depends on the length of the curvature and out‐of‐plane structural distortions at the boundary, having stronger influence the latter one. Moreover, it is found that by increasing the defects at the boundary, the transport properties can strongly be reduced in comparison to the effect produced by heating up the boundary region. This is due to the large reduction of the phonon transmission for in‐plane and out‐of‐plane vibrational modes after increasing the structural disorder in the GBs.

Since its discovery in 2004, graphene, sp^2^‐bonded carbon atoms arranged in a 2D honeycomb lattice, has undoubtedly been one of the most studied low dimensional materials.^1^ Among its remarkable properties,^2^, ^3^ an extremely high thermal conductivity has received great attention both in experiments and calculations. Indeed, to fully exploit the extraordinary promises of this first truly 2D material, the production of large size and good quality graphene layers has to be mastered. One of the most promising routes is the growth of graphene by chemical vapor deposition on various metal surfaces.^4^ However, similar to many other macroscopic materials, graphene exists in polycrystalline structures, with numerous 1D topological defects called grain boundaries (GBs).^5^, ^6^, ^7^, ^8^, ^9^ These GBs have been experimentally shown to be formed by nonhexagonal rings (pentagons, heptagons, nonagons, etc.) among misoriented, but crystallographically perfect, graphene lattices. Moreover, it has been widely proved that the transport properties can be reduced one to two orders of magnitude below those of graphene films.^2^, ^3^, ^10^, ^11^, ^12^, ^13^ Hence, the presence of grain boundaries can have a dramatic impact on the performance of various graphene‐based nanodevices.

In previous studies, phonon scattering for a class of grain boundaries, tilt boundaries, has been addressed using nonequilibrium molecular dynamics (MD)^14^, ^15^, ^16^ and nonequilibrium Green's functions.^17^, ^18^, ^19^, ^20^ Tilt boundaries have mirror symmetry with respect to the boundary, that is, the crystallographic orientations of the grains are different for each GB structure. In this work, our aim is to address the effects of integrity (structure and dynamics) and the geometry (linear and curved,^6^ see **Figure** **1**) of the GB on phonon transport properties. Therefore, we fix the crystallographic orientations of the grains as armchair and zigzag at opposite sides and generate random GBs. In order to achieve this, we start with two seeds with armchair and zigzag orientations, which are ≈10 to 25 Å apart, depending on the target GB geometry. For generating a linear boundary (GB1 and GB2), we start with seeds that have clean linear edges and add carbon atoms one‐by‐one to the available sites. The positions of the added atoms are determined by taking into account the positions of the nearest neighbor atoms. Addition of carbon atoms to random sites continues until the grains converge together. Afterward, excess atoms, which give rise to coordination numbers larger than three, are removed. For generating curved boundaries, we start with edges that have asperities toward each other. The asperities are thin ribbons of graphene. Increasing the length of the asperity, we obtain GBs with larger curvatures (GB7 and GB8). The growth process is carried out so as to fulfill periodic boundary conditions in the transverse direction (*y*‐direction) and spurious effects due to edges are avoided.

**Figure 1:**
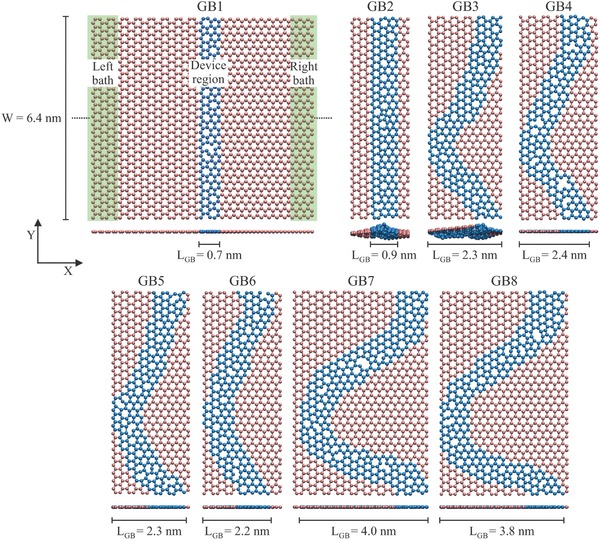
Top and side views of the structures of linear (GB1 and GB2) and curved (GB3–GB8) graphene grain boundaries. *L*_GB_ is the length of the grain boundary. The same width (*W*) has been used for all the systems. For similar *L*_GB_, different defects were considered. For GB1, we also show a schematic representation of the partition scheme for transport calculation using the Green's function technique. GB2 and GB3 display strong out‐of‐plane distortions while the rest of the structures remain almost planar. The carbon atoms in blue color are the atoms mainly forming the grain boundary while the carbon atoms in pink color are the atoms far from the boundary region.

Structural optimizations of GBs have been performed by using density functional tight‐binding (DFTB) method as implemented in the DFTB+ software package.^21^, ^22^ This method combines accuracy with numerical efficiency, and it allows dealing with systems up to 2000 atoms in a quantum simulation, especially for carbon‐based materials.^19^, ^20^, ^23^ Optimizations were performed until the absolute value of the interatomic forces is below 10^−5^ atomic units with a *k*‐point mesh of 1 × 12 × 1. We have considered a width of ≈ 6.4 nm for all the grain boundaries and periodic boundary conditions were imposed along the *y*‐direction. The GB length and curvature are measured by *L*_GB_. Structures with similar *L*_GB_ present different type of nonhexagonal rings.

The phonon transport problem is treated using atomistic Green's function technique (AGFT) as implemented in DFTB+ code (in‐house version). Based on this, a partition of the whole system into left bath, device region, and right bath is carried out and the dynamical matrices, *K*, for each subsystem it is obtained by numerically differentiating the forces calculated using DFTB method. In our study, the device region includes the periodic linear/curved defect (grain boundary), whereas the left and right baths are taken as a unit cell of the 2D graphene grains in each side, i.e., armchair and zigzag, respectively (see Figure 1 for the case of GB1). These grains are considered semi‐infinite along the *x*‐direction based on the Green's function formalism.^24^ Using this information, the retarded phonon Green's function (*G* ^r^) for the central region (including the coupling to the thermal baths through self‐energies) can be calculated, $G^{r}(\omega )={\left(\omega^{2}I-K-\Sigma_{L}^{r}(\omega )-\Sigma_{R}^{r}(\omega )\right)}^{-1}$, with *I* as the identity matrix and Σ_L/R_ being the self‐energy for left (L) and right (R) baths, respectively, which are calculated as described in refs. 24, 25. Having obtained the GF and self‐energy matrices, the phonon transmission spectrum, τ_ph_(ω), can be computed as

(1) $$\tau_{\mathrm{ph}}(\omega )=\mathrm{Trace}\left(G^{r}(\omega )\Gamma_{L}(\omega )G^{a}(\omega )\Gamma_{R}(\omega )\right)$$

where $\Gamma_{\mathrm{L/R}}(\omega )=i[\Sigma_{\mathrm{L/R}}^{r}(\omega )-\Sigma_{\mathrm{L/R}}^{a}(\omega )]$ are the broadening functions and *G* ^a^(ω) = [*G* ^r^(ω)]† is the advanced Green's function. As far as only elastic processes are considered (no anharmonic interactions or coupling to electronic degrees of freedom), this formalism is mathematically similar to the Landauer approach for charge transport. For systems, in which elastic scattering is dominant, AGFT describes phonon transport very accurately.^26^, ^27^ Then, the thermal conductance is defined as

(2) $$\kappa_{\mathrm{ph}}=\frac{1}{2\pi k_{B}T^{2}}\int_{0}^{\infty }{\left(\hbar \omega \right)}^{2}\frac{e^{\hbar \omega /k_{B}T}}{{\left(e^{\hbar \omega /k_{B}T}-1\right)}^{2}}\tau_{\mathrm{ph}}(\omega )d\omega$$

Here, *k*_B_ and *h* are the Boltzmann and the Planck constants, respectively. This expression is obtained by a linear expansion in the applied temperature difference Δ*T* of the quantity *N*_B_(*T* + Δ*T*) − *N*_B_(*T*), where *N*_B_ is the Bose–Einstein distribution. To gain insight of the phonon transport properties, we have computed the in‐plane and out‐of‐plane mode contribution to the total transmission function and thermal conductance, i.e., (τ, κ)_total_ = (τ, κ)_in_ + (τ, κ)_out_. We note that such separation of in‐plane and out‐of‐plane contributions is straightforward whence these modes are already decoupled in the reservoirs, which is the case in graphene.

In **Figure** **2**, the transmission spectra of different GB structures are displayed and they are compared with that of pristine graphene (pink solid line), where transmission over the entire spectrum is lowered as expected due to local structural defects.^19^, ^20^ Moreover, transport channels in linear GBs present higher transmission than those in curved GBs, mainly in the high‐frequency region which corresponds to in‐plane mode contributions. In fact, GB3 has the lowest transmission probability because of its curved structure and the out‐of‐plane distortions (see **Figure** **3**), which strongly reduces τ_in_. In graphene, out‐of‐plane distortions locally couple in‐plane and out‐of‐plane degrees of freedom, which are decoupled otherwise, and this coupling is the main scattering mechanism for phonons in deformed graphene structures.^28^ Consequently, linear GBs show higher values of thermal conductance. Slight differences in κ for GB1 and GB2 are also found, being more pronounced for *T* < 200 K, see the inset in the right panel of Figure 2, where we show *P*_GB_ = κ_GB_/κ_graph_ as a function of the temperature, being κ_GB_ and κ_graph_ the thermal conductance for a given grain boundary and graphene pristine, respectively.^18^ This behavior is related to the reduction of the transmission probability for low‐frequency modes induced by the out‐of‐plane distortions at the boundary. This effect also takes place for GB4, GB5, and GB6, which have similar *L*_GB_ but different thermal conductances. GB6 displays the highest thermal conductance among the three due to the smaller number of structural defects at the boundary. In addition, we found that from all the curved GBs, independently of *L*_GB_, GB3 displays the lowest κ as a result of the large suppression of transmission over the whole frequency range, while GB7 shows the second lowest thermal conductance indicating that the influence of curvature (*L*_GB_) is weaker than the out‐of‐plane structural distortions.

**Figure 2:**
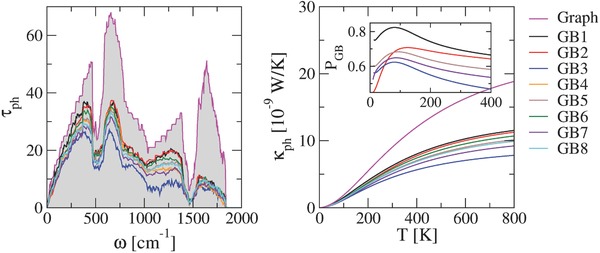
Phonon transmission (left panel) and thermal conductance (right panel) for graphene grain boundaries. The results are compared with the corresponding functions for pristine graphene (labeled as graph). Inset of right panel shows the temperature dependence of *P*_GB_ = κ_GB_/κ_graph_.

**Figure 3:**
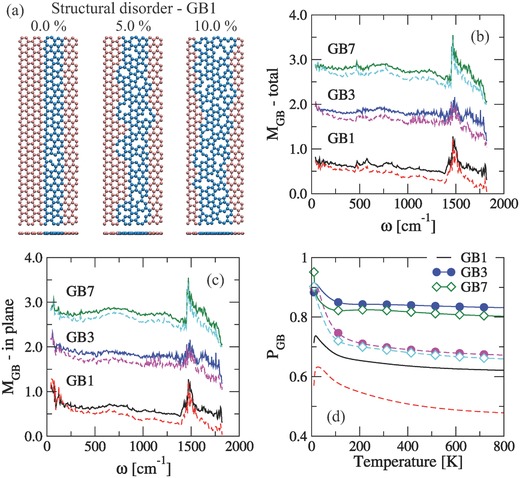
a) Schematic representation of the mechanism for introducing atomic defects (structural disorder) into the boundaries. *M*_GB_ parameter as a function of mode frequency for b) total and c) in‐plane phonon transmission of graphene GBs at different level of atomic defects. d) Temperature dependence of *P*_GB_ parameter. For all the graphs, solid and dashed lines correspond to 5% and 10% of atomic defects, respectively. The high peak around ω ≈ 1500 cm^−1^ in panels (b) and (c) is coming from the small values on the phonon transmission.

Next, the thermal response of graphene GBs after structurally modifying the boundaries has been analyzed. Structural disorder was introduced by randomly removing atoms from the boundary, as depicted in Figure 3a for GB1. All the results are averaged over three random configurations. To gain a better quantitative understanding, we have defined the parameter *M*_GB_ = τ_GB_(%)/τ_GB_(0), where τ_GB_(%) is the phonon transmission for a given percentage of atomic defects and τ_GB_(0) is the initial transmission (i.e., without extra defects). The phonon transmission for both types of GBs is reduced after increasing the percentage of atomic defects,^29^ the effect being more dramatic for linear GBs because of its higher degree of structural order compared to curved GBs (see Figure 3b). This is a result of τ_in_ and τ_out_ being influenced by the atomic defects in different frequency ranges, τ_out_ is the most affected for ω < 200 cm^−1^ while the reduction of transmission probability for in‐plane modes becomes larger at high frequencies ω > 1000 cm^−1^ (see Figure 3c). Additionally, we have computed *P*_GB_ = κ_GB_(%)/κ_GB_(0) to study the variation of the thermal conductance with the level of atomic defects for the GBs (see Figure 3d). Here, GB1 shows the highest reduction of thermal conductance at any percentage of atomic defects. Whereas curved GBs, independent of the *L*_GB_ value, display similar reduction of their corresponding thermal conductance, which is slightly reduced at very low temperatures, *T* < 50 K. The later effect is a consequence of the small variation on τ_ph_ for ω < 200 cm^−1^ where out‐of‐plane modes are dominant.^17^ It has been observed that κ for linear and curved GBs with 5% of atomic defects is reduced by the same factor at *T* > 200 K, i.e., *P*_GB_ remains constant. On the contrary, *P*_GB_ decreases as a function of the temperature at 10%. Moreover, regardless the type of GBs, all of them have very similar thermal conductance at 10% of atomic defects. It is worth mentioning that our findings are in agreement with the recent thermal conductance measurements in single graphene grains done by Yasaei et al.,^9^ where they observed that the thermal conductance per unit area is affected by a complex interplay between the amount of edge roughness and amount of disorder in the disordered patch.

Finally, we have addressed the influence of dynamical disorder produced by coupling a Nose‐Hoover thermostat only to the device region of the transport setup (see **Figure** **4**a). In this sense, the magnitude of atomic displacements around the equilibrium position (i.e., at 0 K) will be controlled by the device temperature *T*_D_. Quantum molecular dynamics simulations were carried out using the DFTB+ code,^30^, ^31^, ^32^, ^33^ i.e., the corresponding total energy is calculated every MD step and the Verlet algorithm is used for the time integration of the Newton's equation of atomic motion with a time step of 0.5 fs. The results are then averaged over 10 configurations which were taken every 0.05 ps after the device region reached the temperature equilibration. All the MD runs were performed until the temperature reached the equilibrium (≈2.5 ps). We have compared the phonon transmission functions obtained at different temperatures, τ_GB_(*T*), with the corresponding zero temperature, τ_GB_(0), by redefining *M*_GB_ = τ_GB_(*T*)/τ_GB_(0).

**Figure 4:**
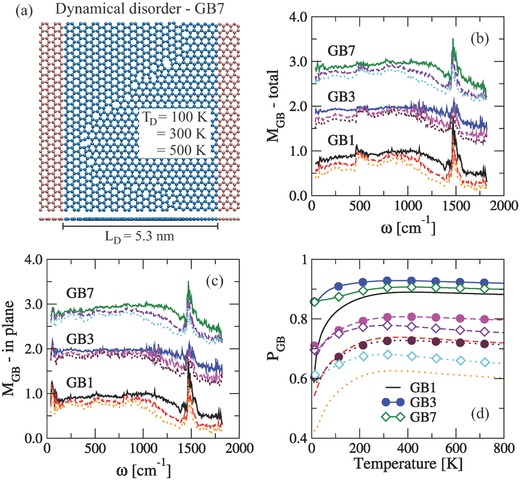
a) Schematic representation of the transport setup to perform the MD simulations. The thermostat is only imposed to the atoms on the device region (blue balls). *M*_GB_ parameter as a function of mode frequency for b) total and c) in‐plane phonon transmission of graphene GBs at different *T*_D_. d) Temperature dependence of *P*_GB_ parameter. For all the graphs, solid, dashed, and dotted lines correspond to *T*_D_ = 100, 300, and 500 K, respectively. The high peak around ω ≈ 1500 cm^−1^ in panels (b) and (c) is coming from the small values on the phonon transmission.

Similar to the influence of structural disorder, the phonon transmission of GBs is reduced when the device temperature *T*_D_ increases (see Figure 4b). However, transmission probability for ω < 1300 cm^−1^ remains almost the same at *T*_D_ = 100 K, i.e., *M*_GB_ ≈ 1.0. For grain boundaries at *T*_D_ = 500 K, the transmission function has been completely altered for low‐frequency (ω < 500 cm^−1^) and high‐frequency (ω > 1000 cm^−1^) modes. The reduction at low‐frequency modes is mainly due to the strong suppression of transfer channels belonging to out‐of‐plane modes since τ_in_ is slightly reduced after increasing *T*_D_ (see Figure 4c). This is different from the effect observed with atomic defects in which both τ_in_ and τ_out_ are strongly affected. It has also been found that the influence of the dynamics is stronger for linear GBs. We now use the parameter *P*_GB_ = κ_GB_(*T*)/κ_GB_(0), to find out the impact of dynamical disorder on κ_ph_ of graphene GBs (see Figure 4d). In fact, it was found that the thermal conductance for linear GBs is the most altered, specially, at low temperature of the leads (*T* < 100 K), *P*_GB_ → 0, showing an opposite behavior to this with atomic defects where κ_ph_ is slightly reduced at this temperature range (*P*_GB_ ≈ 1). This effect is related to small modifications on the in‐plane phonon transmission. GBs with large curvature are more influenced by *T*_D_. Besides, we have that the reduction factor of κ_ph_ for all the GBs at a given *T*_D_ is the same when *T* > 200 K, i.e., *P*_GB_ is invariant. It is worth noting that despite considering very high *T*_D_, the thermal conductance is still larger to those obtained at 10% of structural disorder.

In conclusion, we have used an atomistic Green's functions' approach to systematically study the phonon transport properties of large graphene grain boundaries with a focus on the influence of structural and dynamical disorder. Curved GBs show the lowest thermal conductance, especially when out‐of‐plane distortions are added on the boundary that leads to a reduction of the in‐plane contribution to the transmission (τ_in_). Moreover, structural disorder strongly affects in‐plane and out‐of‐plane mode contributions to the phonon transmission. On the contrary, dynamical disorder only weakly affects τ_in_. Hence, the thermal conductance of linear and curved GBs is reduced by the effect of both types of disorder, the effect being stronger for linear GBs due to their higher degree of structural order. In general, our results show that dynamical effects on the phonon transport properties of graphene GBs have less impact comparing to structural disorder. These findings provide insights into the thermal response of large graphene grain boundaries with different geometries that have been successfully grown through several experimental techniques.

## Conflict of Interest

The authors declare no conflict of interest.
